# Evaluation of the cutaneous sensation of the face in patients with different clinical forms of leprosy

**DOI:** 10.1371/journal.pone.0213842

**Published:** 2019-03-14

**Authors:** Marlice Fernandes de Oliveira, Douglas Eulálio Antunes, Diogo Fernandes dos Santos, Isabela Maria Bernardes Goulart

**Affiliations:** 1 Postgraduate Program on Health Sciences, School of Medicine, Federal University of Uberlândia (UFU), Uberlândia, Minas Gerais, Brazil; 2 University Center of Cerrado, Patrocínio, Minas Gerais, Brazil; 3 National Reference Center for Sanitary Dermatology and Leprosy, University Hospital, School of Medicine, Federal University of Uberlândia (UFU), Uberlândia, Minas Gerais, Brazil; University of Würzburg, GERMANY

## Abstract

**Background:**

Leprosy can be considered to be the most common peripheral neuropathy of infectious etiology and constitutes a public health problem. The standard routine examination for assessing sensory impairment in leprosy neuropathy basically evaluates hands, feet and eyes. However, evaluation of facial cutaneous sensation is not routinely performed.

**Objectives:**

The aim of this study was to evaluate facial cutaneous sensation in patients with different clinical forms of leprosy and compare the findings with those from healthy individuals.

**Methodology:**

19 healthy controls and 71 leprosy patients who were being treated at a national reference center for leprosy in Brazil underwent facial sensation assessment using the Semmes-Weinstein monofilament test. This test was applied over the facial areas corresponding to the ophthalmic, maxillary and mandibular distal branches of the trigeminal nerve.

**Results:**

The predominant clinical form in terms of changes to facial cutaneous sensation was lepromatous leprosy (LL), followed by the borderline-borderline (BB), and borderline-lepromatous (BL) forms, in comparison with healthy individuals. The distal branches most affected were the zygomatic (28.2%; 20/71), buccal (23.9%; 17/71) and nasal (22.5%; 16/71). There was asymmetrical sensory impairment of the face in 62.5% (20/32) of the cases.

**Conclusion:**

The face is just as impaired in leprosy as are the feet, hands and eyes, but facial impairment is underdiagnosed. Our evaluation on the different sensory branches and evidence of asymmetrical impairment of the face confirm the classically described pattern of leprosy neuropathy, i.e. consisting of asymmetrical and predominantly sensory peripheral neuropathy.

## Introduction

Leprosy is a chronic bacterial disease for which the etiological agent is *Mycobacterium leprae* (*M*. *leprae)*. The long incubation period and the insidious symptoms and signs of leprosy make it difficult to diagnose. Although leprosy is classically recognized as a dermatological disease, it is primarily a neurological disease. It constitutes a public health problem, mainly due to its incapacitating potential and the strong social discrimination and stigma that are associated with this disease [1,2].

Leprosy is classified into five clinical forms, according to the host's immune response, the histopathological classification of the cutaneous lesion and the bacillary load. Patients with a better cellular immune response (mediated by T lymphocytes) are classified as having tuberculoid (TT) leprosy, while anergic patients with a humoral response are classified as having lepromatous (LL) leprosy. Patients between these two extremes are defined as borderline cases, presenting intermediate immune responses, with three subdivisions: borderline-tuberculoid (BT), borderline-borderline (BB) and borderline-lepromatous (BL) [3,4,5]. Primary neural leprosy, also known as pure neural leprosy, is another clinical form in which there is clinical evidence of peripheral neuropathy together with absence of skin lesions and negative findings from slit-skin smear bacilloscopy [2,6].

The chronic course of this disease is modified by acute inflammatory processes called leprosy type 1 and 2 reactions. These result from changes to the immune balance between the host and *M*. *leprae* that lead to increased morbidity and impairment of peripheral nerve function [7, 8, 9].

Involvement of the peripheral nerves is present in all clinical forms of leprosy, usually as asymmetrical peripheral neuropathy that is predominantly sensory. The peripheral nerves most commonly affected are the ulnar, median, common fibular, tibial, cutaneous radial, facial and major auricular nerves [2,6,10,11]. However, only a few studies have evaluated facial sensation in leprosy and impairment of the trigeminal nerve [12,13,14].

The global strategy for leprosy control that was proposed for the period 2016–2020 places emphasis on early diagnosing of cases prior to the emergence of visible disabilities [15]. Therefore, evaluation of cutaneous sensation in leprosy plays a fundamental role in diagnosing peripheral nerve impairment, with the aim of avoiding progressive and permanent loss of function, since sensory alterations precede motor alterations [2,16].

The standard routine examination for assessing sensory impairment in leprosy neuropathy in Brazil makes use of Semmes-Weinstein monofilaments, and basically evaluates hands and feet. The cutaneous surface of the face is not included in this routine, except for the ophthalmic branch of the trigeminal nerve [17].

The aim of the present study was to evaluate the cutaneous sensation of the face in patients with different clinical forms of leprosy and compare this with facial sensation in healthy individuals.

## Material and methods

### Subjects and type of study

This was a cross-sectional observational study in which 71 leprosy patients and 19 healthy controls were selected. In the leprosy group, the following distribution according to clinical form was observed: 13 TT, 14 LL, 22 BT, 10 BB and 12 BL.

This study considered eligible patients who had been diagnosed with leprosy and were undergoing treatment at a national reference center for leprosy in Brazil over the period from 2014 to 2016. Patients who showed other possible etiologies for peripheral neuropathies and those who were presenting a reaction episode at the time of the evaluation were excluded.

**Fig 1:**
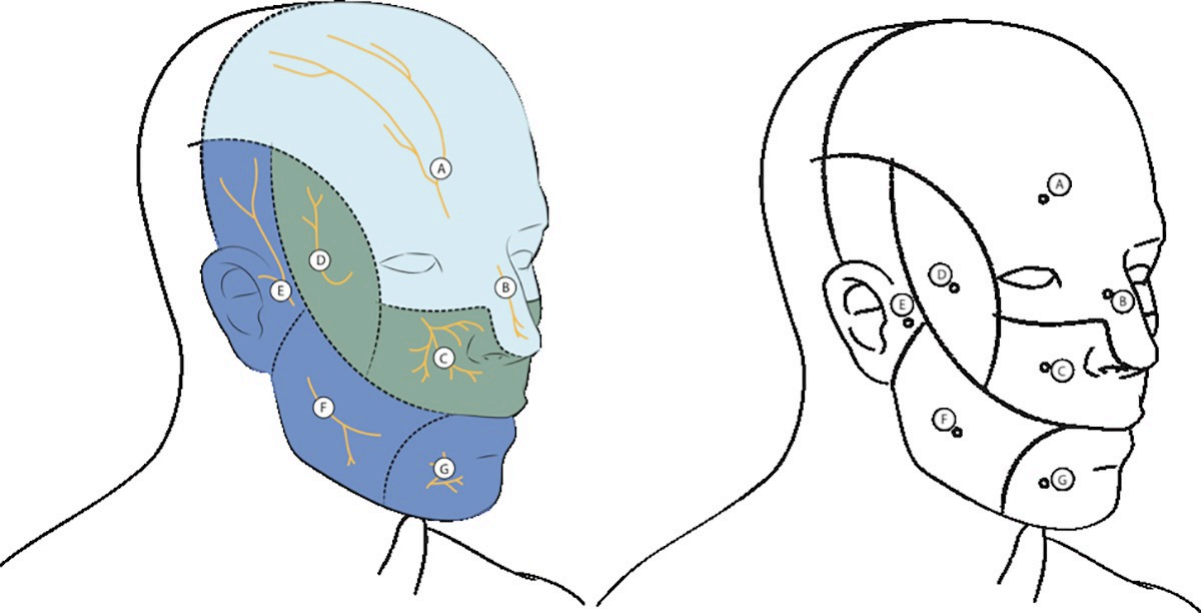
Assessment of facial sensation according to the anatomical distribution of the trigeminal nerve branches: A- supraorbital, B- nasal, C- infraorbital, D- zygomatic, E- auriculotemporal, F- buccal and G- mental.

**Fig 2:**
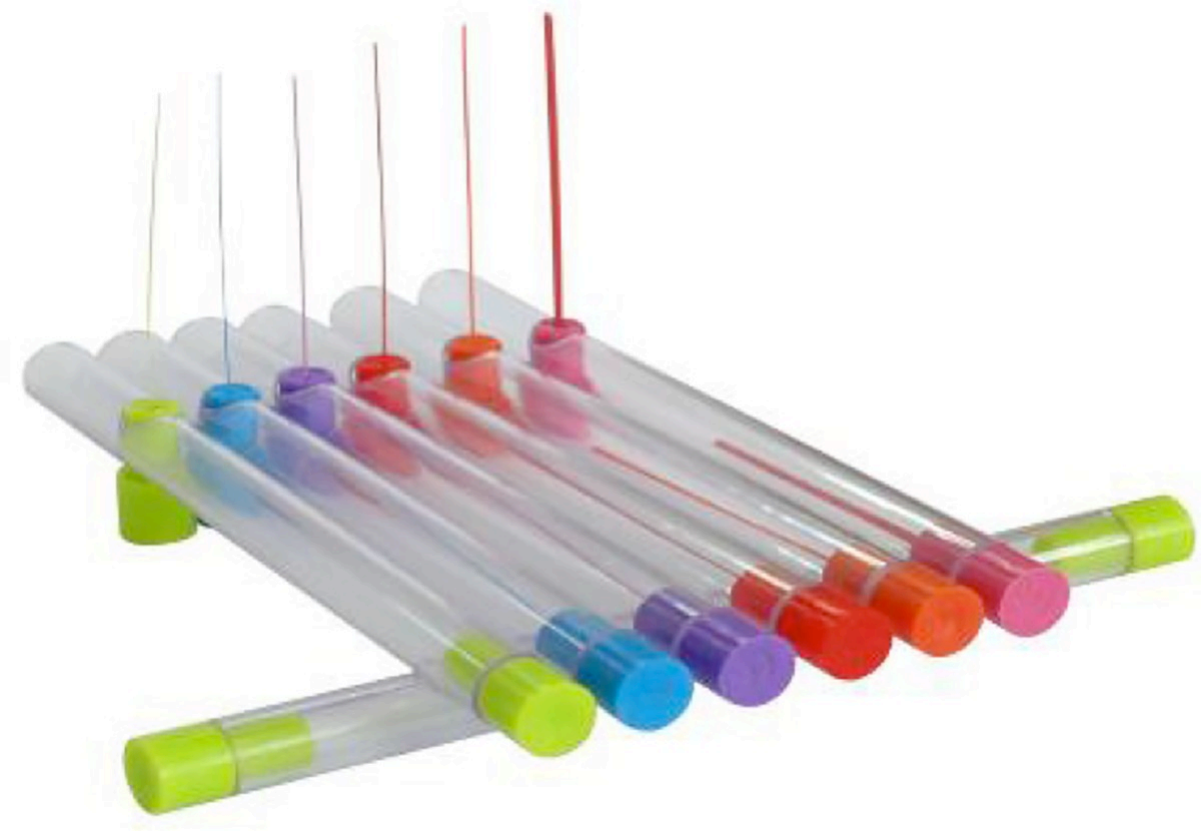
(A) Semmes-Weinstein esthesiometer with six nylon monofilaments of different diameters: 0.05 g (green), 0.2 g (blue), 2 g (purple), 4 g (red), 10 g (orange) and 300 g (pink).

The dermatoneurological evaluation and facial sensation assessment were performed by a single expert professional. The face was subdivided into seven regions, according to the anatomical distribution of the trigeminal nerve branches: supraorbital, nasal, infraorbital, zygomatic, auriculotemporal, buccal and mental (Fig 1). The sensitivity level of each nerve was evaluated using the six Semmes-Weinstein filaments, which exert forces of 0.05 g, 0.2 g, 2 g, 4 g, 10 g and 300 g on the skin (Fig 2). The perception of 0.05 g on the face was considered normal, while facial sensitivity to forces greater than this was considered altered. In the presence of a negative response to the lightest monofilament (0.05 g), the test was carried out using monofilaments of increasing thickness, i.e. 0.2 g, 2 g, 4 g, 10 g and 300 g, until a positive response was obtained [17].

The level of facial functional disability was evaluated in accordance with the protocol recommended by the Brazilian Ministry of Health, which only evaluates ocular alterations in the face. In grade 1, decreased eyelid muscle strength is observed, without visible deficiencies and/or decreased or lost corneal sensitivity, but with delayed response or absence of blink reflex. In relation to facial grade 2 disability, observations were based on the presence of visible deficiencies, such as lagophthalmos, ectropion, entropy, trichiasis, central corneal opacity, iridocyclitis, inability to count fingers at six meters or visual acuity < 0.1 or 6:60, excluding other causes [17].

### Ethics statement

The Ethics Committee of the Federal University of Uberlandia approved the study (CAAE: 41933614.3.0000.5152.). Written informed consent was obtained from all participants.

### Statistical analysis

Fisher’s exact test was used to compare the control group (healthy individuals) and the groups with the various clinical forms of leprosy (TT, BT, BB, BL and LL) regarding the proportions of individuals with changes to cutaneous sensitivity of the face. Fisher’s exact test was also used to compare the proportions of changes to the symmetry of cutaneous sensitivity of the face (left and right). For all analyses, we used GraphPad Prism version 7 (GraphPad Software Inc., San Diego, CA, USA) with an alpha significance level of 5% (0.05).

## Results

Seventy-one patients who had been diagnosed with leprosy and nineteen healthy controls were included in this study between 2014 and 2016. None of the comparisons of epidemiological characteristics between the groups showed any significant difference.

The clinical and epidemiological characteristics of the leprosy group are described in Table 1. It is important to emphasize that, regarding the degree of facial disability at diagnosis, only 1.4% (1/71) presented disability grade 1 and 1.4% (1/71) had disability grade 2.

**Table 1:** Clinical-epidemiological data on leprosy patients.

Variables	N	%
**Clinical form**		
TT	13	18.31
BT	22	30.99
BB	10	14.08
BL	12	16.90
LL	14	19.72
**Sex**		
Male	42	59.16
Female	29	40.84
**Age group**		
20–39	19	26.77
40–59	36	50.70
≥ 60	16	22.53
**Degree of ocular disability**		
0	69	97.20
1	1	1.4
2	1	1.4

In the control group, no individual presented sensory impairment on the face. In the leprosy groups, 45% (32/71) of the patients presented at least one abnormality. The individuals most affected were those of the LL group (85.7%; 12/14) followed by BB (80%; 8/10), BL (50%; 6/12), BT (22.7%; 5/22) and TT (7.7%; 1/13).

The LL group presented very significant impairment in the facial sensory evaluation (p < 0.0001), in comparison with the healthy group, and these patients also presenting the highest sensory threshold on the face (300 g). The BB and BL groups also presented significant facial sensory impairment, compared with the control group (Table 2).

**Table 2:** Evaluation of cutaneous sensation of the face in the different clinical forms of leprosy.

Healthy participants and leprosy groups	Abnormal		Normal		Total number of individuals	Higher sensory threshold	P_1_	P_2_	Fisher’s test
	n	%	n	%	(n = 90)				*p-value*
Healthy	0	0	19	100	19				
T	1	7.7	12	92.3	13	0.2 g	Healthy	T	0.4062
BT	5	22.7	17	77.3	22	4.0 g	Healthy	BT	0.0507
BB	8	80	2	20	10	10.0 g	Healthy	BB	<0.0001
BL	6	50	6	50	12	2.0 g	Healthy	BL	<0.0013
LL	12	85.7	2	14.3	14	300.0 g	Healthy	LL	<0.0001

Table 3 presents separate analysis on each trigeminal branch. The percentage of sensory deficit in the face of patients with leprosy, ordered according to degree of impairment, was as follows: 28.2% (20/71) in the zygomatic branch, 23.9% (17/71) in the buccal; 22.5% (16/71) in the nasal; 16.9% (12/71) in the infraorbital; 15.5% (11/71) in the auriculotemporal; and 14.1% (10/71) in the frontal.

**Table 3:** Abnormalities in cutaneous sensation of the face in the different clinical forms of leprosy, according to the distal branch of the trigeminal nerve (Fisher's exact test).

Cutaneous sensitivity of the face								
Healthy participants and leprosy groups	**Abnormal**		**Normal**		**Fisher’s exact test**			
	Frontal branch							
	N	%	n	%	N total	p_1_	p_2_	p-value
Healthy	0	0	19	100	19			
TT	0	0	13	100	13	Healthy	TT	-
BT	2	9.1	20	90.9	22	Healthy	BT	0.4902
BB	2	20	8	80	10	Healthy	BB	0.1108
BL	2	16.7	10	83.3	12	Healthy	BL	0.1419
LL	4	29	10	71	14	Healthy	LL	0.0245
	Nasal branch						
Healthy	0	0	19	100	19			
TT	0	0	13	100	13	Healthy	TT	-
BT	3	13.6	19	86.4	22	Healthy	BT	0.2354
BB	5	50	5	50	10	Healthy	BB	0.0021
BL	2	16.7	10	83.3	12	Healthy	BL	0.1419
LL	6	43	8	57	14	Healthy	LL	0.0027
	Infraorbital branch							
Healthy	0	0	19	100	19			
TT	0	0	13	100	13	Healthy	TT	-
BT	2	9.1	20	90.9	22	Healthy	BT	0,4902
BB	2	20	8	80	10	Healthy	BB	0,1108
BL	1	8.3	11	91.7	12	Healthy	BL	0,3871
LL	7	50	7	50	14	Healthy	LL	0.0008
	Zygomatic branch							
Healthy	0	0	19	100	19			
TT	0	0	13	100	13	Healthy	TT	-
BT	3	13.6	19	86.4	22	Healthy	BT	0.2354
BB	5	50	5	50	10	Healthy	BB	0.0021
BL	3	25	9	75	12	Healthy	BL	0.0489
LL	9	64	5	36	14	Healthy	LL	0.0001
	Auriculotemporal branch							
Healthy	0	0	19	100	19			
TT	0	0	13	100	13	Healthy	TT	-
BT	2	9.1	20	90.9	22	Healthy	BT	0.4902
BB	2	20	8	80	10	Healthy	BB	0.3448
BL	0	0	12	100	12	Healthy	BL	-
LL	7	50	7	50	14	Healthy	LL	0.0008
	Buccal branch							
Healthy	0	0	19	100	19			
TT	1	7.7	12	92.3	13	Healthy	TT	0.4062
BT	3	13.6	19	86.4	22	Healthy	BT	0.2354
BB	5	50	5	50	10	Healthy	BB	0.0021
BL	1	8.3	11	91.7	12	Healthy	BL	0.3871
LL	7	50	7	50	14	Healthy	LL	0.001
** **	Mental branch							
Healthy	0	0	19	100	19			
TT	0	0	13	100	13	Healthy	TT	-
BT	3	13.6	19	86.4	22	Healthy	BT	0.2354
BB	1	10	9	90	10	Healthy	BB	0.3448
BL	0	0	12	100	12	Healthy	BL	-
LL	2	14.3	12	85.7	14	Healthy	LL	0.1723

In the evaluation on the frontal branch, 14% (10/71) of the patients presented a sensory deficit in this region. Only the clinical form LL differed from the healthy group (p = 0.0245). In the nasal branch, 22.5% (16/71) presented some abnormality, predominantly in the LL (0.0027) and BB (0.0021) clinical forms, compared with the control group (Table 3).

In the infraorbital branch, 16.9% (12/71) presented a sensory deficit, and LL patients were the only ones significantly different from the healthy group (p = 0.0008) (Table 3). In comparing the clinical forms with each other, the LL group (58.3%; 7/12) presented a significant difference in relation to TT (p = 0.0058), BT (p = 0.0144) and BL (0.0357), in separate evaluations on this branch.

Abnormalities in the zygomatic branch were demonstrated in 28.2% (20/71) of the cases, especially in the clinical forms LL (p = 0.0001), BB (p = 0.0021) and BL (p = 0.0489), compared with the healthy group (Table 3). In comparisons between the clinical forms, the clinical form LL was statistically different only from the BT form (p = 0.0032).

It was observed that 14% (10/71) of the patients presented changes in the auriculotemporal branch, and that only the LL form differed from the healthy group (p = 0.0008) (Table 3), and that this form differed from the clinical form BT (p = 0.0144).

The evaluation on the buccal branch showed abnormalities in 23.9% (17/71) of the patients, and significant differences between the healthy group and two of the clinical forms: LL (p = 0.001) and BB (p = 0.0021) (Table 3). Comparison between the clinical forms showed that LL (41%; 7/17) was statistically different from TT (p = 0.0329) and from BT (p = 0.0262).

The mental branch did not present any statistically significant change.

In evaluating symmetry, 31.2% (10/32) presented unilateral facial involvement and 68.8% (22/32) presented bilateral impairment. However, among the patients with bilateral impairment, 45.5% (10/22) presented different degrees of impairment in the sensory evaluation. Therefore, there was asymmetrical sensory impairment of the face in 62.5% (20/32) of the cases.

## Discussion

The present study characterized sensory evaluations of the faces of 71 patients with diagnoses of leprosy who were assisted at a national reference center for leprosy in Brazil. This study highlighted the importance of this approach for prevention of disabilities.

Leprosy is an infectious disease that primarily compromises peripheral nerves. The pattern of neural involvement consists of asymmetrical multiple mononeuropathy that is predominantly sensory but also can cause motor and even autonomic impairment. In addition, leprosy neuropathy usually presents characteristic temperature-dependent impairment that relates to the preference of the bacillus for colder areas. Therefore, the face is often compromised in this disease. Nonetheless, the face is not routinely evaluated in referral services for patients with leprosy [2,6,12].

The degree of sensory impairment of the face in the patients with leprosy in the present study was considerable. However, there are few studies in the literature that corroborate these findings [12]. It is important to note that disabilities are considered to include functional alterations that may impede not only patients’ social inclusion but also their activities of daily living. Such alterations contribute negatively to these patients’ quality of life, consequent to the sensory, motor or autonomic disorders that develop in their hands, feet, eyes and face [15].

Ophthalmological evaluation is highly important in relation to leprosy, since the ocular alterations that occur in leprosy are responsible for severe disabilities that may lead to loss of independence among these individuals and may constitute a potential threat to self-care. Several studies have already described the main ophthalmological changes relating to leprosy [18,19,20,21]. However, in the routine evaluation method currently used for assessing the degree of disability, the face is not adequately evaluated, as we showed in our study, in which few patients presented impairment according to their routine assessments of disability, while almost half of them already presented sensory impairment.

Regarding neural involvement in leprosy cases, we observed this in all clinical forms of leprosy, although there were important differences in its presentation and severity and in the extent of impairment, depending on the individual's immune response. It should be emphasized that these changes can occur before, during or even after the specific treatment for the disease. The consequences from peripheral neural impairment form the main cause of sequelae and disability in leprosy. There is still great difficulty among healthcare professionals regarding recognition of neural abnormalities, since cutaneous manifestations are better known and more easily recognized in clinical practice [22,23,24,25].

In this study, the clinical forms LL, BB and BL were most severely affected, in comparison with the control group. Impairment of the peripheral nerves was seen to occur more diffusely in these patients, with multiple nerves affected by the bacillus. Despite the extent of the disease, the host’s inflammatory response was weak, thus relatively preserving the architecture and function of the nerves, even in the more advanced stages of the disease. These data mainly reflected the delay between the onset of symptoms and making the diagnosis, since facial changes in leprosy neuropathy may progress slowly and progressively, even in the absence of reactional episodes, as a silent neuropathy [2,26,27].

Our evaluation on the different sensory branches and evidence of asymmetrical impairment of the face confirm the classically described pattern of leprosy neuropathy, i.e. consisting of asymmetrical and predominantly sensory peripheral neuropathy [2].

## Conclusion

Sensory changes to the face are of great importance in personal care, hygiene and orofacial functions. These activities are carried out with great automatism and require adequate and accurate sensitivity information for them to be carried out safely. Lack of sensitivity poses a risk of trauma or abrasion through lowering of the body's protection.

The face is just as impaired in leprosy as are the feet, hands and eyes, but facial impairment is underdiagnosed. Although not involved in labor or economic activities, facial changes may compromise individuals’ social integration. Facial impairment in leprosy is often responsible for low self-esteem, a sense of rejection and perpetuation of the stigma related to this infection.
